# Skeletal age prediction model from percentage of adult height in children and adolescents

**DOI:** 10.1038/s41598-020-72835-5

**Published:** 2020-09-25

**Authors:** Luis Alberto Flores Olivares, Lidia G. De León, Maria Isabel Fragoso

**Affiliations:** 1 Facultad de Ciencias de La Cultura Física, Universidad Autónoma de Chihuahua, Chihuahua, México; 2 Laboratory of Physiology and Biochemistry Exercise, CIPER, Faculdade de Motricidade Humana, Universidade de Lisboa, Estrada da Costa, 1499-002 Cruz-Quebrada, Dafundo Portugal

**Keywords:** Bone development, Paediatric research

## Abstract

Skeletal age (SA) is considered the gold standard to assess the degree of maturation and has been widely used in sports, education and public health areas; however, it requires sophisticated equipment and well-trained technicians. Therefore, it is important to develop non-invasive methods for its evaluation. The aim was to develop an equation to predict SA using the percentage of adult height. SA was measured by Tanner-Whitehouse-3 method, and the percentage of adult height was estimated by two methodologies: Tanner-Whitehouse-3 method (P-TW3) and Khamis-Roche method (P-KR) using 839 schoolchildren of both sexes. Linear regression was used for predicting SA from P-TW3; then P-TW3 was replaced in the equation for P-KR value. Bland–Altman graphs, interclass correlation coefficient and Kappa index were used as validation tests. Model showed a SA predictive capacity of 93.2% in boys and 96.8% in girls. The average differences between SA measured and SA predicted by P-TW3 was 0.0504 (± 0.664) in boys and 0.0144 (± 0.435) in girls (P = 0.229 and 0.667, respectively). When P-TW3 was replaced for P-KR value in the equation, the average differences were − 0.0532 in boys and 0.0850 in girls (P = 0.509 and 0.167 respectively). The present model, based on the percentage of adult height, showed an adequate estimation of SA in children and adolescents and it can be used in the absence of bone X-ray equipment, in healthy boys aged 9 to 15 and girls 8 to 13.

## Introduction

Biological maturation assessment, which refers to the process of becoming mature^1^, provides the possibility to observe the interindividual variations on magnitude (development level), timing (onset of specific changes) and tempo (rate of change) in children and adolescents^2^; and has been used to identify sports talents^3^, to analyse associations between physical aspects and motor skills performance^4^, to obtain more accurate data on overweight and obesity during childhood^5^ and to identify metabolic risks during puberty^6^.

Several methodologies have been mentioned in past published literature to determine the biological maturation degree, commonly classified through three indicators: (1) bone maturity, gives us the skeletal age (SA), according to skeletal tissue development; (2) sexual maturity, refers to the set of changes in sexual characteristics, associated with reproductive capability; and (3) somatic maturity, indicates the growth rate of body dimensions (height is commonly used), where the traditional assessment ways are: the age at peak height velocity (PHV) and prediction of adult stature, from which the percentage of adult height is estimated^2^. A maturation indicator is an event that must comply with the following conditions: (a) reflect changes in a biological system; (b) occur in all individuals as they progress to adulthood; (c) reach the same final stage in all individuals; (d) show continuous progress in such a way that discrete stages can be identified in a continuum; (e) be applicable throughout the organic maturation process; (f) be independent of body growth^7^.

SA (bone maturity) and percentage of adult height (somatic maturity) are maturity indicators that reflect the stage of maturity and the attained growth rate; however, amongst the available biological maturity indicators normally used in infants, children, and adolescents, SA remains the gold standard^2,8^. All children have a skeleton made up of cartilage and progress to a fully ossified adult skeleton. The events regarding bone development are invariable; all bones mature in the near-distal direction and show the same sequence of maturational events, regardless of the social and biologic conditions of the participants. Skeleton maturation can be obtained from the bones of the hand and wrist^9–12^, although other body segments, such as cervical vertebrae^13–15^, knee, hip and foot, can be also used^7^. The most common methods for SA assessment through hand–wrist radiography are the Greulich & Pyle^11^, the Tanner & Whitehouse^16^ and the Fels method (Roche et al., 1988)^17^.

In 1962, Tanner, Whitehouse and Healy proposed a new methodology for assessing bone maturity (TW1), with a more solid mathematical structure. To access bone maturity a radiograph of the left hand and wrist was necessary. Each hand and wrist bone were observed separately according to three main concepts: (1) the number of ossification centres present; (2) the level of ossification of each bone and; (3) the number of epiphyseal fusions occurred^16^, and to each bone was assigned a stage (A to I or H) and a score. The bone classifications vary, with the progression of the development stage, from A (initial stage) to I (final or mature stage) for most of the bones. In 1975, a revision of this methodology (TW2) was published. The scores attributed to each stage were improved to be in accordance with specific maturation processes of carpal bones, radius, ulna and short bones and also with sex differences^18^. These methodological changes allowed the estimation of adult height and percentage adult height.

The most recent version of Tanner Whitehouse method—TW3^12^, included only 13 bones and was developed from a broader and more diversified sample which included British, Belgian, Italian, Spanish, Argentine, North American and Japanese children. The bones observed were: radius, ulna, metacarpals (I, III and V), proximal phalanges (I, III and V), medium phalanges (III and V) and distal phalanges (I, III and V), and the obtained score is named the RUS (radio, ulna and short bones) score. This method has been considered the most objective among the methods that use bone indicators^19^. Skeletal maturity provides a reasonably accurate and reliable estimation of biological age, can be applied throughout the postnatal maturation period and allows, if the evaluator knows the subject's height, prediction of adult height. However, it requires sophisticated equipment, exposure to a low level of radiation, great level of proficiency from the evaluator and a constant quality control, since this evaluation by stages is actually a continuous process^1,8^.

The percentage of adult height is a somatic maturity indicator that requires the estimation of skeletal maturation^12,20^, at least for the most accurate systems. Roche–Wainer–Thissen (RWT)^20^ method has been considered to be the most accurate method to predict percentage adult height for children who do not have important pathological conditions and is based on linear regression including variables such as: average height of biological parents, current height, body mass and bone age determined by the Greulich–Pyle method^21^. However, it is also possible to estimate maturity without using skeletal age^21,22^. Based on a modification of the RWT method, aiming at simplifying it by excluding bone age, the Khamis–Roche method^21^ was developed studying white healthy North American children (223 boys and 210 girls) participating in Fels Longitudinal Study, using the following variables: current height, body mass, chronological age and average height of biological parents, adjusted for sex. This method is applicable to children from 4.0 to 17.5 years of age, being the most accurate for predicting percentage adult height without including bone age^23^. Percentage of adult height attained at the time of observation, is a non-invasive and easy to use method, especially the procedure developed by Khamis and Roche in 1994^21^. This indicator of maturation has been used on studies of sports, motor skills, physical activity and exercise physiology^23–27^. However, there are few studies on non-invasive estimation methods^28^, and to the best of our knowledge, only one study in the past has predicted bone age based on anthropometric variables^29^.

The establishment of non-invasive, low-cost and easy-to-use methods to estimate SA is ever more relevant considering the growth importance of maturity in the area of physical activity. Thus, in this context the aim of the present study was to develop a methodological strategy to predict SA, based on the percentage of adult height, in children and adolescents.

## Methods

The present non-experimental, prospective, cross-sectional and correlational study was authorized by the Faculty of Human Kinetics of Lisbon University Ethics Committee and implemented according to Helsinki Declaration^30^.

Participants were readily available and were not designated by means of a statistical criterion^31^. Three thousand adolescents of three schools, ages between 10 and 17 years, received an informed consent form to participate in a school-based cross-sectional study covering several issues. The schools were chosen by their geographical distribution covering three of the nine municipalities of the Greater Lisbon (Oeiras, Lisboa, Loures), Portugal. A sample of 1008 schoolchildren was recruited, although 149 did not meet the inclusion criteria. Children with muscular, bone or joint impediments; a skeletal age ± 3 years from decimal age; incomplete data (ex: parents’ height) or physical/mental disabilities, were not included. The present study included 839 adolescents (497 boys from 9 to 15 years and 342 girls from 8 to 13 years) with a mean age of 12.0 (SD = 1.58 years) e 10.38 (SD = 2.21 years), respectively. All subjects granted their consent of voluntary participation and an informed consent was signed by their parents/guardians. SA was measured by wrist-hand radiography in all school children and percentage of adult height was estimated by two different methodologies.

### Measures

Skeletal age (SA). Biological maturity was assessed through SA evaluation according to the Thaner–Whithouse III method^12^ by two trained examiners. A left hand–wrist radiograph was taken using a portable X-ray model Ascor 110, which operates with a low level of radiation (set to 3 mA/s and 36 kV or 5 microsieverts). Subsequently, radiograph films were developed at the Faculty of Human Kinetics of Lisbon University, with a Gevamatic 60 film processing machine, using AGFA G-153 developer and G-354 fixer fluids. Mean differences of SA assessments was 0.03 years (± 0.04) and the inter-observer technical error measurement was 0.12 years.

Anthropometric measures. Body mass and stature were measured according to international Society for the Advancement of Kinanthropometry guidelines^32^. Body mass was measured with a Secca body scale, model 761 7019009 with a sensitivity of 0.5 kg; and stature was measured with a Siber–Hegner anthropometric kit with a sensitivity of 0.1 cm. Both measurements were made in duplicate by two anthropometric technicians, level 2 ISAK. The subjects wore shorts and T-shirts, shoes were removed. The intra-observer technical error of weight measurement was null; and 0.29 cm for stature. Those errors were considered adequate.

Adult height prediction. Two methodologies were used to estimate adult height: (1) the method developed by Tanner et al., in 2001 (TW3)^12^ which uses as variables the subject height, in centimetres and the scores obtained from a left-hand-wrist X-ray; and (2) the procedure developed by Khamis and Roche in 1994 (KR)^21^, which uses as predictive variables, the height in inches, weight in pounds, chronological age and the average height of the parents in inches, which is self-reported. Once the predicted adult height was attained by both protocols, the percentage of adult height achieved at the time of observation was calculated for all subjects (P-TW3 and P-KR).

### Procedure

The sample was randomly divided into two groups. The first group consisted of 245 boys and 173 girls; and was used to generate SA prediction equations from P-TW3 (independent variable). This methodological strategy was built to identify the coefficients used in the predictive equations, considering that SA and P-TW3, obtained through the same method, have a very high correlation^12^; then P-TW3 was replaced by P-KR as predictor variable, assuming that P-TW3 and P-KR methods are not statistically different, as observed in a previously published study^33^. To estimate differences between TW3 and KR methods Flores and collaborators applied in a previous study One ANOVA, Bland–Altman graphs as well as a Kruskal–Wallis test. No differences were found in percentage adult height estimated with TW3 and KR methods, in all age groups and in both sexes, when subjects were classified by chronological age (P > 0.05). Likewise, no secular changes were observed in percentage adult height (P > 0.05) showing that percentage adult height is a very constant and stable maturity indicator throughout time. In the present study, differences between these two methodologies were also contrasted. These previous results led us to consider that P-TW3 and P-KR would be interchangeable.

Second group, consisted of 252 boys and 169 girls, was used to validate SA prediction equations with P-TW3 and; to validate SA prediction equations with P-KR, only 208 boys and 169 girls were used because the stature of parents needed to estimate Khamis–Roche percentage adult height^21^ was not available for the 44 remaining boys.

SA was used as numerical and categorical variable classified in three different categories according to the difference between SA and chronological age: late, on time or early maturers. On time when the difference was within ± 1.0 year of chronological age; late maturers when SA is less than the chronological age by more than 1.0 year; and early maturers when SA is greater than chronological age by more than 1.0 year^35^.

### Statistical analysis

Statistical analyses were performed using SPSS (version 21, IBM corp.). All statistical tests were performed at 95% confidence level. Normality test was applied by Kolmogorov–Smirnov test in SA measure and TW3 and KR methods, by sex. All variables showed a normal distribution (P > 0.200).

Means and standard deviations of weight, height, chronological age, SA, P-TW3 and P-KR were determined by group 1 and 2 (model and validation groups) and by sex, for all 497 (group 1 = 252; group 2 = 245) boys and 342 (group 1 = 173; group 2 = 169) girls recruited.

A simple linear regression was used on the first group to estimate SA, from the P-TW3; and the linear regression model assumptions, such as linearity, normality and homoscedasticity were determined. Linearity was performed by a simple regression analysis between predictor variable (percentage of adult height) and response variable (SA); the residuals normality was established by the Q–Q plot and Kolmogorov–Smirnov test; homoscedasticity was observed through a scatter plot between residuals and predicted values.

To validate the new SA prediction model using SA obtained by TW3, the following statistical tests were performed: (1) Interclass correlation coefficient was determined to identify the association between SA predicted by TW3 and SA measured; (2) the degree of agreement was evaluated by Bland–Altman graph, establishing the mean value of the differences and the average of both methods; and the limits of agreement as the mean of differences ± 1.96 SD of the difference; (3) Kappa index was obtained to define the level of concordance between SA categories: the gold standard method (SA measured) and the predictor method (SA estimated), for both sexes. A kappa index value lower than 0.40 indicates fair agreement, 0.41 to 0.60 be considered moderate agreement, 0.61–0.80 indicates substantial agreement, and more than 0.81 be considered almost perfect agreement^35^.

A hypothesis test was carried out on the second group to identify the differences between P-TW3 and P-KR using a student t-test.

Finally, another reliability analysis was completed between SA observed and SA predicted, substituting in the equation P-TW3 for P-KR value, applying interclass correlation coefficient and Bland–Altman plot, by sex.

### Ethical approval

The present research was authorized by the Faculty of Human Kinetics of Lisbon University Ethics Committee and implemented according to Helsinki Declaration.

### Informed consent

Informed consent was obtained from all individual participants included in the study.

## Results

### Group 1: model development

The means and standard deviation of group 1 for SA, chronological age, P-TW3, weight, and height by sex, are showed on Table 1.

**Table 1:** Characteristics of group 1 (design of model) subjects by sex.

Variable	Boys				Girls			
	N	Mean	±	SD	N	Mean	±	SD
SA	245	11.4	±	2.08	173	10.38	±	2.21
Chronological age	245	12.0	±	1.58	173	10.67	±	1.41
P-TW3	245	84.7	±	5.84	173	87.24	±	5.97
Weight	245	42.6	±	11.11	173	38.74	±	10.45
Height	245	150.8	±	11.71	173	143.14	±	10.06

*SA* skeletal age, *SD* standard deviation, *P-TW3* Percentage of adult height by TW3 method.

Firstly, β0 and β1 coefficient values (intercept and slope) from linear regression were determined by the relationship between SA, as response variable and P-TW3, as predictor variable. The correlation among those methodologies (SA and P-TW3) was high, 0.96 in boys and 0.98 in girls (P = 0.001). Table 2 shows the characteristics of the SA prediction models based on the P-TW3 by sex. Prediction equations were: boys SA = (− 17.754) + (0.344 * P-TW3); and girls SA = (− 21.311) + (0.363 * P-TW3). Model predictive capacity in boys was 93.2% with an adjusted R square = 0.931; and for girls was 96.8% with an adjusted R square = 0.968 (P = 0.001).

**Table 2:** Models summary for the prediction of skeletal age from percentage of adult height by P-TW3 method, by sex.

Boys					Girls				
R	R^2^	Estimated error	Mean of residuals	Sig	R	R^2^	Estimated error	Mean of residuals	Sig
0.965	0.932	0.554	0.0000001	0.001	0.984	0.968	0.396	0.0000001	0.001
		B	Typical error	Sig			B	Typical error	Sig
Intercept		− 17.754	0.507	0.001	Intercept		− 21.311	0.443	0.001
Coefficient P-TW3		0.344	0.006	0.001	Coefficient P-TW3		0.363	0.005	0.001

*P-TW3* Percentage of adult height by TW3 method.

Linear regression assumptions for both equations were fulfilled. Linearity was verified by the relationship between the predictor variable and the response variable; homoscedasticity was confirmed by the constant variance of residuals for all the predicted values, without finding outliers; and according to the Q–Q plot of normality and Kolmogorov–Smirnov test, normality of residuals was observed (P = 0.20 and 0.051 for boys and girls respectively) Appendix A.

### Group 2: model validation

Means and standard deviation in group 2, for SA measured, SA predicted by P-TW3 and P-KR, chronological age, percentage of adult height by both protocols, weight and height by sex, are showed in Table 3.

**Table 3:** Characteristics of group 2 (validation of model) subjects by sex.

Variable	Boys				Girls			
	N	Mean	±	SD	N	Mean	±	SD
SA	252	11.6	±	2.18	169	10.6	±	1.82
SA by P-TW3	252	11.5	±	2.13	169	10.6	±	1.95
SA by P-KR	208	11.5	±	1.95	169	10.5	±	1.91
Chronological age	252	12.0	±	1.56	169	10.9	±	1.33
P-TW3	252	85.1	±	6.19	169	87.9	±	5.36
P-KR	208	85.0	±	5.66	169	87.7	±	5.27
Weight	252	43.9	±	11.72	169	39.6	±	10.23
Height	252	150.7	±	12.33	169	144.5	±	9.89

*SA* skeletal age, *SD* standard deviation, *P-TW3* Percentage of adult height predicted by TW3 method, *P-KR* Percentage of adult height predicted by KR method.

No differences were found between the two percentages of adult height (P-TW3 and P-KR), both in boys and girls (P = 0.519 and 0.111 respectively) rather showing a high correlation between procedures (P-TW3 and P-KR) [0.976 in boys and 0.956 in girls (P = 0.001)]. Additionally, ANOVA test by Bonferroni post-hoc were applied to compare SA obtained by gold standard and SA obtained through the predictive methods (P-TW3 and P-KR) and no differences were found (P = 0.981 in boys and 0.907 in girls).

**Figure 1:**
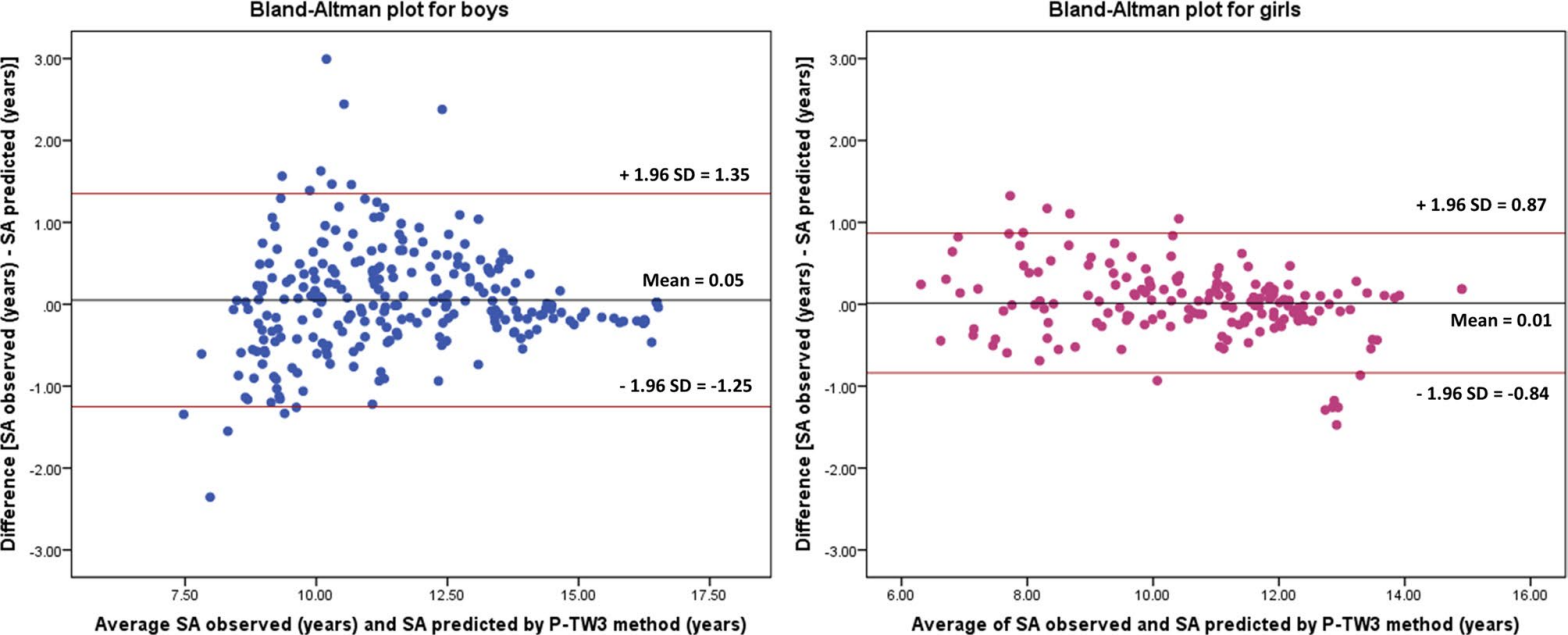
Bland–Altman plots of SA observed, and SA predicted by P-TW3 in the model validation group, by sex.

Reliability analyses between SA measured and SA predicted by P-TW3 showed a high interclass correlation coefficient, 0.976 in boys and 0.986 in girls respectively (P = 0.001). Additionally, according to Bland–Altman analyses (Fig. 1), the average of the differences regarding the two methods was 0.0504 (± 0.664) for boys and 0.0144 (± 0.435) for girls being similar to zero (P = 0.229 and 0.667, respectively). The 95% of boys and 93% of girls were within agreement limits and were within an acceptable range (− 1.250, 1.351 for boys and − 0.838, 0.867 for girls). Also, an adequate Kappa index of 0.7 in boys and 0.8 in girls (P = 0.001) was achieved when comparing the SA status classification (late, on time, early) obtained from the gold standard and from the new model, showing a good agreement between maturity classifications. Table 4 shows percentage concordance between SA observed and SA predicted by P-TW3 method.

**Table 4:** Crosstabulation of maturation status between SA observed and SA predicted by P-TW3 method.

Sex	Maturity status based on SA observed	Maturity status based on SA predicted by P-TW3 method				Percentage accordance
		Late	On time	Early	Total	
Boys	Late	68	19	0	87	
	On time	12	117	2	131	
	Early	0	16	18	34	
	Total	80	152	20	252	81%
Girls	Late	36	6	0	42	
	On time	5	99	5	109	
	Early	0	2	16	18	
	Total	41	107	21	169	89%

*SA* skeletal age, *P-TW3* Percentage of adult height by Tanner-Whitehouse 3 method.

**Figure 2:**
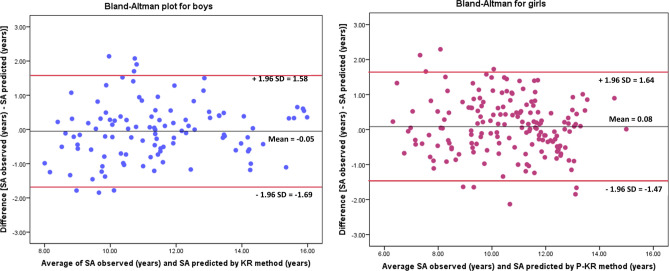
Bland–Altman plots of SA observed, and SA predicted by P-KR in the model validation group, by sex.

**Figure 3:**
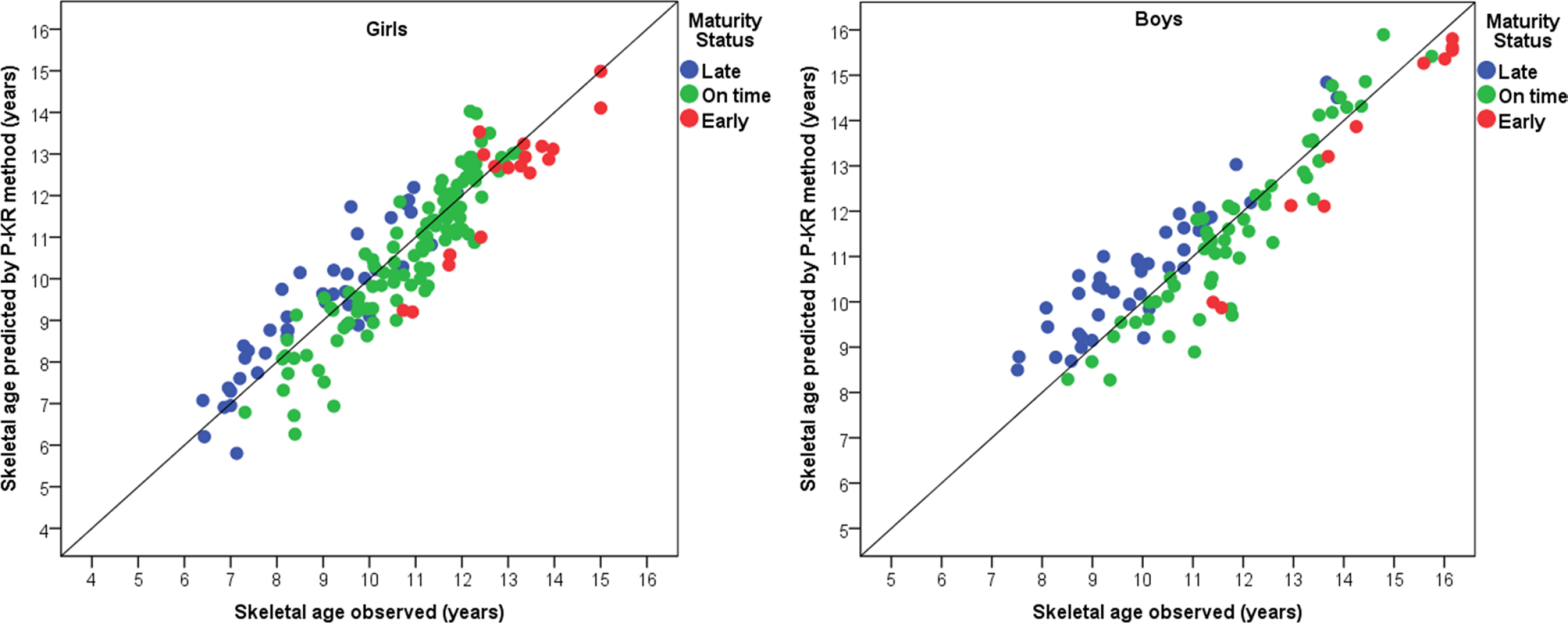
Linear association between SA observed and SA predicted by the P-KR method, by sex.

Finally, when P-TW3 value was replaced by P-KR value in the predictive equation, intraclass correlation coefficient, between SA measured and SA predicted using P-KR, remained high, with a value of 0.955 in boys and 0.952 in girls (P = 0.001). The mean average of the differences between the two methodologies was − 0.0532 in boys and 0.0850 in girls which was similar to zero in both sexes (P = 0.509 and 0.167 respectively). The Bland–Altman plot showed that 93% of boys and 95% of girls were within the agreement limits (− 1.688, 1.582 in boys and − 1.474, 1.644 in girls) and within an adequate range (Fig. 2). Also, a graph showing a linear association between SA observed and SA predicted by the P-KR method, in boys and girls, was presented (R^2^ = 0.84 and 0.83 respectively, p < 0.001), in the Fig. 3. When comparing the maturity status classifications (late, on time, early) obtained from SA gold standard and SA predicted by P-KR, a Kappa index of 0.4 in boys and 0.5 in girls (P = 0.001) was achieved, which represented a fair-moderate agreement between the above maturity classifications methods. Table 5 shows concordance percentage between SA observed and SA predicted by P-KR method.

**Table 5:** Crosstabulation of maturation status between SA observed and SA predicted by P-KM method.

Sex	Maturity status based on SA observed	Maturity status based on SA predicted by P-KR method				Percentage accordance
		Late	On time	Early	Total	
Boys	Late	23	17	0	40	
	On time	10	47	0	57	
	Early	0	8	3	11	
	Total	33	72	3	108	68%
Girls	Late	26	16	0	42	
	On time	14	88	7	109	
	Early	0	8	10	18	
	Total	40	112	17	169	73%

*SA* skeletal age, *P-KR* Percentage of adult height predicted by Khamis–Roche method.

## Discussion

Maturity assessment in epidemiological studies or in large samples is always a great challenge to accomplish. In recent years, a growing need to generate practical and non-invasive methods for predicting maturity has been observed; however, the literature in this field is still very limited^29,36^.

The methodological strategy of the present study, which consists of predicting SA from the percentage of adult height by linear regression was based on a two-step process. Firstly, the model coefficients were established using the P-TW3 method as a predictor variable, since P-TW3 is based on the skeletal age obtained through the Tanner-Whitehouse III method, minimising the prediction error.

Once the prediction model was established, the second-step was to replace P-TW3 value for P-KR value, which was just possible because both protocols assumed identical height percentage results, and thus the variable percentage adult height interchangeable. Also, P-KR protocol is easy to estimate because it uses simple variables, such as height, weight, chronological age of the subject and the average height of parents.

The P-KR method, used in the present study, as predictor variable of SA, is a practical method that is widely employed as a maturation indicator and has already been validated with SA in American adolescents^34^. In addition, P-KR protocol has also shown, through longitudinal studies, a high capacity in predicting the percentage of adult height when comparing it with the reached real values, demonstrating adequate stability^37^.

This new methodological strategy to predict SA, utilizing P-KR protocol, is a non-invasive method and an adequate alternative; it does not require sophisticated equipment; neither requires highly trained personnel and can be used in studies using large samples. Furthermore, the present prediction model fulfilled all linear regression assumptions and disclosed a high association with SA measured by X-ray, considered as the gold standard, showing a high predictive capacity for both sexes.

The prognostic capability of this new methodology is much higher than the one reported by Cabral et al.^29^; who developed the only equation available to estimate SA from anthropometric variables (height, triceps skinfold, corrected relaxed arm girth and humeral and femoral diameters) and chronological age of Brazilian children and adolescents. The R square value reported in the above-mentioned study was 0.754, being so far, the only acknowledged study to present a SA prediction equation from anthropometric variables.

However, Cabral et al.^29^ study, did not demonstrate concordance between the SA maturity status classifications (early, on time, late) obtained through the observed and predicted methods. This study, as far as we know, is the first non-invasive method to estimate skeletal age and present the agreement degree between the maturity status. Skeletal age of advanced children, with this new methodology, tends to be slightly underestimated and that of late maturers is somewhat overestimated; however, a moderate agreement was observed, and 68% of boys and 73% of girls were adequately classified. Similar results have been reported by Malina et al.^34^, that properly classified 61% of boys and reached a moderate agreement between observed skeletal age and a non-invasive estimate of biological maturity status, expressed as a percentage of predicted mature height. Therefore, the SA estimated by the present model can be comfortably used to associate biological age to other numerical variables, but it must be used with caution to classify (late, on time and early) participants into contrasting maturity groups. More studies are necessary to explore the degree of agreement between maturity status, obtained through non-invasive methods, and the gold standard.

Other studies, have been carried out in recent years to estimate SA from linear model equations. Caldas et al.^38^ developed a linear model based on the height of the third and fourth cervical vertebras; however, they did not report the R^2^ of the model. Türkoz et al.^28^ created a similar model, exhibiting high SA correlations, also without specifying the predictive capacity of the model.

Recent studies have also been validating ultrasound and dual-energy X-ray absorptiometry (DEXA) to estimate skeletal age. These alternatives expose the subject to lower and non-ionizing radiation compared to X-ray radiography^39,40^; however, those alternatives require sophisticated equipment at a high cost, which render its use less attractive in epidemiological studies.

The SA evaluation has been considered an important variable within sport sciences related areas, particularly in the study of physical performance^41^, physical activity levels obtained by accelerometry^42^, and in the study of motor skills^43^; in health related areas, such as hyperinsulinemia levels of overweight children^44^, body mass index classification of overweight and obese boys and girls^45^, injury profile of children and adolescents^46^, and in health-related quality of life of children and adolescents^31^; as well as in the education field in its relation to academic performance^47^ These wide SA application supports the importance of the present model.

Within all maturity indicators used to evaluate and establish growth rates, SA is considered the best, not only because of its precision but also because it allows to classify children better than other traditionally used methods. Subjects of similar chronological age may present different maturity status. In this sense, the knowledge of SA parameters is crucial as it allows us to organize children and adolescents according to their biological development, already recognized of great use in health, paediatrics, sports and physical activity domains^8^.

Inter-individual variability in biological maturation is an important issue and absolutely essential during the whole process of talent identification, selection and to the development of training protocols^48^. Ostojic et al.^49^, observed in a longitudinal study that subjects classified biologically as late maturers, at the age of 14 years, achieved a higher elite level than those classified as early maturers.

Due to the importance of maturation, it is of paramount importance to continue this type of research, in order to generate and validate ever better methods. The present equation can be added to other indirect procedures proposed by literature, such as, the estimation of peak height velocity, a timing indicator for somatic maturation^34,50–53^, or the percentage of adult height^22,54–56^.

The development of non-invasive, inexpensive and easy to measure techniques to estimate SA, are proven to be very relevant and necessary in physical activity sciences, especially when measuring large dimensions’ samples. Studies carried out on athletes, equally have shown successful and identical results, when using either invasive or non-invasive methods, which indicate the importance to consider non-invasive methods, especially when SA is concerned^34^.

However, it is important to recognize some limitations associated with the current study. Firstly, the results are limited to the area of Lisbon, so they did not represent the general Portuguese adolescent population. Also, parent statures were self-reported and possibly affected by measurement errors. Additionally, children and adolescents who played sports were not specifically considered in this study as it was previously done by Malina et al.^34^. Finally, it is recommended to use these equations with caution in non-Portuguese samples as Portuguese population may have some somatic and proportional differences in relation to other populations. A broader investigation must be further considered with a more representative sample, considering both rural and urban areas, and with more specific samples considering different type of sports.

In conclusion, the present study describes a new methodological strategy to predict SA from P-KR in boys, 9 to 15 years and in girls, 8 to 13 years. This methodology shows to estimate SA, within the agreement limits, in 93% of boys and 95% of girls and that classifies appropriately the maturation levels. In addition, these results suggest, that this newly developed equation is reliable, practical, non-invasive and can be used in the absence of bone radiology equipment and X-rays, mainly in studies where a large number of participants take part.

## Supplementary information

Supplementary file1
