# Supplementary material for: Skeletal age prediction model from percentage of adult height in children and adolescents
Source: Sci Rep. 2020 Sep 25;10:15768. doi: 10.1038/s41598-020-72835-5 (PMC7519670; doi:10.1038/s41598-020-72835-5)
Supplement: Supplementary file 1 — Supplementary file1 [file 41598_2020_72835_MOESM1_ESM.pdf]

Title Page

Skeletal age prediction model from percentage of adult height in children and adolescents.

1. Luis Alberto Flores Olivares[1]. lolivares@uach.mx
2. Lidia G De León[1]. gdeleon@uach.mx
3. *Maria Isabel Fragoso[2]. ifragoso@fmh.utlisboa.pt

[1]Facultad de Ciencias de la Cultura Física. Universidad Autónoma de Chihuahua. Chihuahua, México.

[2]Laboratory of Physiology and Biochemistry Exercise, CIPER, Faculdade de Motricidade Humana, Universidade de Lisboa, Cruz-Quebrada, Dafundo, Portugal

***Correspondence author:**

Isabel Fragoso, PhD in Human Movement,

Professor of Growth Maturation and Physical Performance and Kinanthropometry,

Laboratory of Physiology and Biochemistry of Exercise,

CIPER, Faculdade de Motricidade Humana, Universidade de Lisboa, Estrada da Costa, 1499-002, Cruz-Quebrada, Dafundo, Portugal,

Phone number: +351 914019110, Fax number: +351 214151248,

ifragoso@fmh.ulisboa.pt

Appendix A.

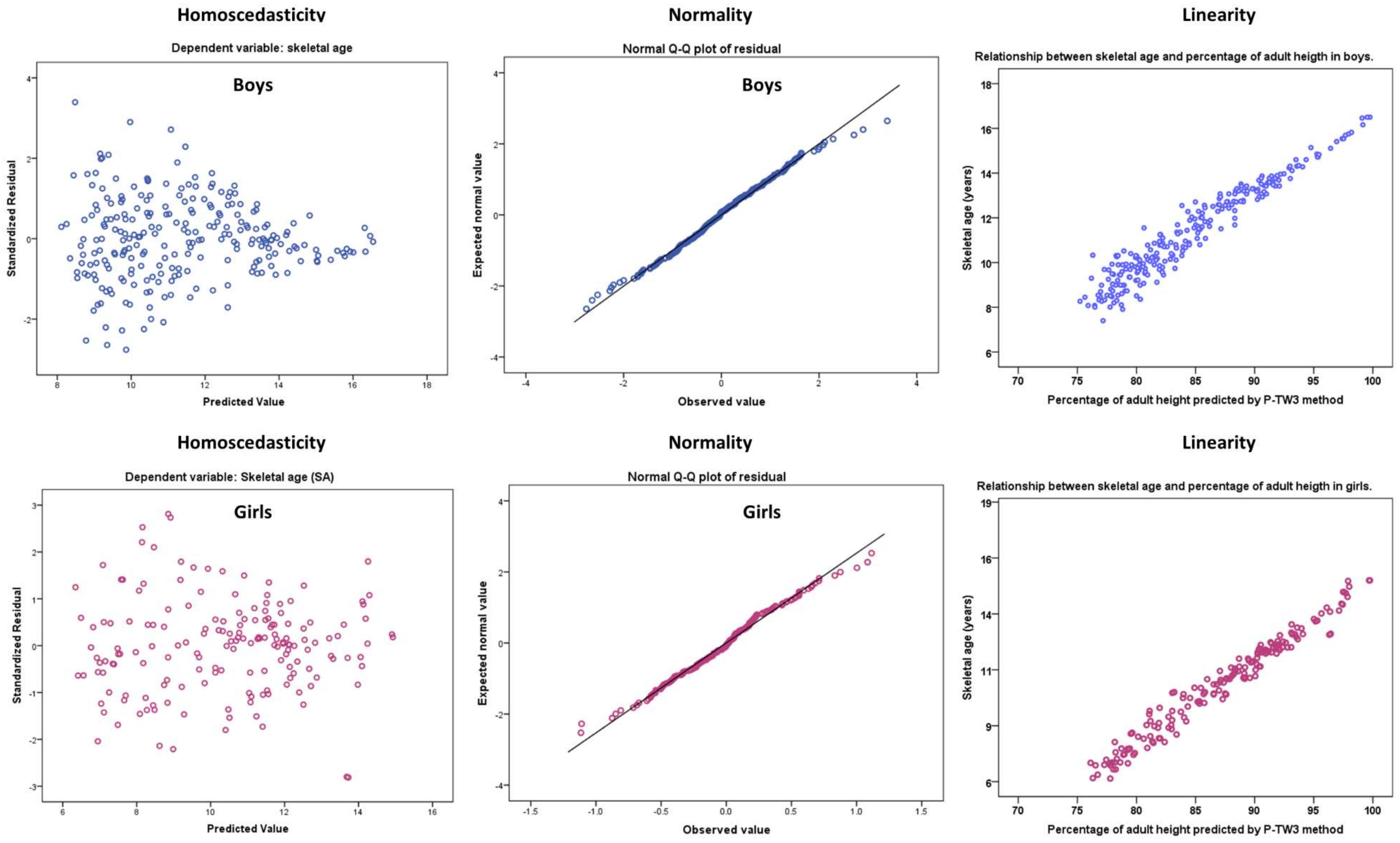

Linear regression assumptions in the model development group, in boys and girls.
